# Dietary *Lycium barbarum* Polysaccharide Modulates Growth Performance, Antioxidant Capacity, and Lipid Metabolism in Common Carp (*Cyprinus carpio*) Fed with High-Fat Diet

**DOI:** 10.3390/antiox13050540

**Published:** 2024-04-28

**Authors:** Di Wu, Jinnan Li, Ze Fan, Zhipeng Sun, Xianhu Zheng, Haitao Zhang, Hong Xu, Liansheng Wang

**Affiliations:** 1 Key Laboratory of Aquatic Animal Diseases and Immune Technology of Heilongjiang Province, Heilongjiang River Fisheries Research Institute, Chinese Academy of Fishery Sciences, Harbin 150070, China; wudi@hrfri.ac.cn (D.W.); lijinnan@hrfri.ac.cn (J.L.); fanze@hrfri.ac.cn (Z.F.); sunzhipeng@hrfri.ac.cn (Z.S.); 2 Key Laboratory of Aquatic, Livestock and Poultry Feed Science and Technology in South China, Ministry of Agriculture and Rural Affairs, Guangdong Evergreen Feed Industry Co., Ltd., Zhanjiang 524000, China; lh0888@163.com; 3 College of Life Science, Huzhou University, Huzhou 313000, China; xuhongtt@126.com

**Keywords:** *Lycium barbarum* polysaccharide, common carp (*Cyprinus carpio*), growth performance, antioxidant capacity, lipid metabolism

## Abstract

To investigate the ameliorative effects and mechanism of *Lycium barbarum* polysaccharide (LBP) on growth performance, oxidative stress, and lipid deposition in common carp (*Cyprinus carpio*) fed with high-fat diets, fish with an initial weight of 5.29 ± 0.12 g were divided into five experimental groups—including normal-fat diets, high-fat diets, and high-fat diets—supplemented with LBP (0.5, 1.0, and 2.0 g/kg) for 8 weeks. The results showed that high-fat diets resulted in significant decreases in final body weight, weight gain rate, and specific growth rate of fish, as well as causing a significant decrease in hepatic total antioxidant capacity, catalase, and glutathione peroxidase activities. These changes were accompanied by a significant decrease in lipase activity and ATP level and a significant increase in malondialdehyde content. The expression levels of lipid metabolism-related genes (acetyl coenzyme A carboxylase 1, stearoyl coenzyme A desaturase 1, fat synthase, peroxisome proliferator-activated receptor-γ, fructofuranose bisphosphatase, and glucose-6-phosphatase) were also markedly elevated by high-fat diets. Supplementation with 0.5–2.0 g/kg LBP in high-fat diets improved the reduced growth performance, increased hepatic total antioxidant enzymes, catalase, and glutathione peroxidase activities, and lowered malondialdehyde level in fish fed with high-fat diets. Additionally, dietary supplementation with LBP significantly downregulated hepatic gene expression levels of acetyl coenzyme A carboxylase 1, stearoyl coenzyme A desaturase 1, fat synthase, sterol regulatory element-binding protein 1, peroxisome proliferator-activated receptor-γ, fructofuranose bisphosphatase, and glucose-6-phosphatase. In conclusion, fish fed with high-fat diets demonstrated impaired growth performance, antioxidant capacity, and lipid metabolism, and dietary supplementation with 0.5–2.0 g/kg LBP ameliorated the impairments induced by high-fat diets.

## 1. Introduction

In intensive aquaculture, it is common to adopt the strategy of increasing the lipid level in diets to conserve protein to achieve the objectives of saving production costs, reducing nitrogen discharge, and protecting the aquaculture environment [1]. However, the demand and ability of fish to utilize lipid is limited by their physiological conditions. Excessive lipid level in diets often leads to lipid accumulation, causing lipid metabolism obstacles and disorders in fish, resulting in nutritional fatty liver and reduced growth performance [2]. More seriously, it also causes oxidative stress damage, decreases disease resistance, and even causes violent deaths of fish, which seriously restricts the healthy and sustainable development of the aquaculture industry [3]. Studies have confirmed that excessive lipid is capable of causing disorders in hepatic lipid synthesis and metabolism, resulting in an imbalance between hepatic lipid synthesis and lipid oxidation [1,4]. Furthermore, high lipid levels can also cause oxidative stress in fish, which ultimately leads to liver injury [5]. Therefore, it has become a hot research topic to regulate the lipid metabolism of fish through green feed additives to reduce lipid deposition and enhance anti-oxidative stress ability.

*Lycium barbarum* polysaccharide (LBP) is a water-soluble polysaccharide extracted from *Lycium barbarum*, including glucose, mannose, and galactose, which is essentially a sugar-protein polyme [6]. LBP has a variety of physiological functions such as immune regulation, hypoglycemia and hypolipidemia, and inhibition of lipid peroxidation [7]. Therefore, LBP is also considered as one of the most important active ingredients in *Lycium barbarum*. In recent years, LBP has been extensively studied in model organisms. Studies have reported that dietary supplementation of LBP significantly improved digestive enzyme activity, antioxidant capacity, and muscle amino acid composition [8,9]. In addition, studies have shown that LBP can ameliorate liver injury and oxidative stress in the organism caused by lipid deposition [10,11,12]. Previous studies have demonstrated that LBP effectively scavenges free radicals such as O_2_^−^ and OH^−^, inhibits malondialdehyde formation, and delays the formation of superoxide in the liver [13,14]. It has been reported that LBP was able to reduce lipid deposition by inhibiting the expression level of PPAR-γ [15]. Apart from that, LBP has also been demonstrated to upregulate LPL protein expression and activate PPAR-α and AMPK pathways [16], and the effects of LBP on these pathways or genes may be the key to its hypoglycemic and hypolipidemic effects. Currently, studies on LBP in aquatic animals are limited. Dietary supplementation with appropriate levels of LBP promoted growth and improved immune function in Turkestan barbel (*Luciobarbus capito*) [17], spotted sea bass (*Lateolabrax maculatus*) [18], hybrid grouper (*Epinephelus lanceolatus* ♂ × *E. fuscoguttatus* ♀) [19], and grass carp (*Ctenopharyngodon idellus*) [20]. It was also found that supplementation with LBP had a protective preventive effect against apoptosis and inflammation in Nile tilapia (*Oreochromis niloticus*) [21,22]. However, studies on LBP in aquatic animals have mainly focused on its effects on growth promotion and disease resistance, whereas studies on its effects on lipid metabolism in aquatic animals have not yet been conducted. It is believed that LBP has a great potential for application in fish fed with high-fat diets and the problem of lipid deposition in aquatic animals, and the specific action mechanism of LBP is also worthy of further investigation.

Common carp is one of the widely farmed species in China. Common carp possesses excellent traits and is subject to high market demand in China [23]. Because of its high essential amino acid and protein contents, it is popular among the public. The production of carp farming in China reached 2.84 million tons in 2022 [24]. At present, the protective effect of LBP on carp under high-fat diet has not been reported. Therefore, in this study, different levels of LBP were supplemented into high-fat diets to investigate the effects of LBP on growth performance, antioxidant capacity, and lipid metabolism of common carp under high-fat diets, which would be beneficial for the rational application of LBP in high-fat diets for common carp.

## 2. Materials and Methods

### 2.1. Materials

The LBP (polysaccharides contents ≥ 51.5%) was purchased from Shanghai Yuanye Biotechnology Co., Ltd. (Shanghai, China). The total antioxidant capacity (T-AOC, CAS No.: A015-2-1), catalase (CAT, CAS No.: A007-1-1), glutathione peroxidase (GSH-Px, CAS No.: A006-2-1), malondialdehyde (MDA, CAS No.: A003-1-2), and lipase (LPS, CAS No.: A054-2-1) kits were obtained from Nanjing Jiancheng Biotechnology Co., Ltd. (Nanjing, China). The fatty acid synthase (FAS, CAS No.: ml062342), malic enzyme (ME, CAS No.: ml025924), glucose 6-phosphate dehydrogenase (G6PD, CAS No.: ml063862), and ATPase (CAS No.: ml063943) kits were supplied by Shanghai Mlbio Biotechnology Co., Ltd. (Shanghai, China). The 6-phosphogluconate dehydrogenase (6PGD, CAS No.: JLC12319) kit was purchased from Shanghai Jingkang Biotechnology Co., Ltd. (Shanghai, China).

### 2.2. Methods

#### 2.2.1. Experimental Diets

Fishmeal, chicken meal, soybean protein concentrate, and soybean meal were used as protein sources, while fish oil and soybean oil were used as lipid sources to formulate the basal (NL) and high-fat (HL) diets. The lipid content of NL and HL was 5% and 15%, respectively. Fish in LBP-treated groups were fed with the high-fat diet supplemented with LBP at a dosage of 0.5 g/kg (recorded as 0.5 g/kg LBP), 1.0 g/kg (recorded as 1.0 g/kg LBP), and 2.0 g/kg (recorded as 2.0 g/kg LBP), respectively. The composition and ingredients of the five diets are shown in Table 1. Briefly, the raw materials were crushed and mixed step by step after passing through a 60-mesh sieve and then extruded to pelletize the pellets, which were dried and sealed at 60 °C and stored at −20 °C for subsequent use.

#### 2.2.2. Feeding Experiment

The feeding experiment was conducted in an indoor recirculating water aquaculture system at the Heilongjiang Fisheries Research Institute, Chinese Academy of Fisheries Sciences. The experimental fish were acclimatized with the basal diet for 2 weeks and fasted for 24 h. After that, 450 healthy carp (initial weight of 5.29 ± 0.12 g) were selected, randomly divided into five groups, and kept in culture tanks with a volume of 288 L (0.6 m length × 0.6 m width × 0.8 m height). Each group had three replicates of 30 fish in each tank. The experimental fish were fed at 8:30, 12:30, and 16:30 daily for 8 weeks. The fish were fed at a fixed daily rate of 5% BW/days for the whole trial period, the weight of all carp in the tank was measured every 14 days during the trial period, and the feeding rate was adjusted according to the weight. The water temperature and pH were maintained at 24 ± 1.0 °C and 7.6 ± 0.4, respectively, and one-third of the water in the tank was changed every day to ensure the survival conditions of the experimental fish.

#### 2.2.3. Sample Collection

At the termination of feeding trial, samples were collected after fasting for 24 h. Six fish were randomly selected in each tank and anesthetized with MS222. The fish were dissected on ice, and liver samples were collected from the same area. Immediately thereafter, liver samples with a thickness of about 2 mm were placed into 2.5% glutaraldehyde phosphate buffer and stored at 4 °C for transmission electron microscopy. The remaining liver samples were immediately stored in liquid nitrogen and then transferred to −80 °C refrigerator for storage for subsequent enzyme activity analysis and RNA extraction.

#### 2.2.4. Calculation of Growth Performance

At the end of the feeding trial, the total weight of the fish in each tank was weighed separately, and the number of fish was recorded for calculating the average weight of the fish in each tank. The parameters of growth performance, including weight gain rate (WGR), feed conversion ratio (FCR), and specific growth rate (SGR), were calculated as follows:

WGR = (final body weight − initial body weight)/initial body weight × 100%.

SGR = (ln final body weight − ln initial body weight)/feeding days × 100%.

FCR = wet weight gain (g)/dry feed intake (g) × 100%.

#### 2.2.5. Biochemical Analysis

T-AOC, CAT, GSH-Px, MDA, and LPS were determined according to the procedure described in the instructions of the commercial kits. The 2,2′-azinobis-(3-ethylbenzthiazoline-6-sulfonic acid) diammonium salt (ABTS) method was used to measure T-AOC, and the Coomassie brilliant blue method was used to measure protein concentrations in liver. Liposynthetic enzyme (FAS, ME, G6PD, and 6PGD) and ATP levels were measured using ELISA method following the manufacturer’s instructions.

#### 2.2.6. Quantitative Real-Time PCR Analysis

The hepatic total RNA was extracted using an RNAiso Plus kit (Takara, Dalian, China). After determining the concentration and purity of the total RNA, cDNA was synthetized using a PrimeScriptTM RT reagent Kit (Takara, Dalian, China). Finally, quantitative real-time PCR was performed using an ABI 7500 real-time PCR System (ABI, Applied Biosystems, Waltham, MA, USA) with SYBR Green (Takara, Dalian, China), which was described previously [5]. The primers sequences are shown in Table 2. After normalization against the reference gene β-actin, the relative levels of mRNA expressions were calculated according to the 2^−∆∆Ct^ method.

#### 2.2.7. Transmission Electron Microscopy Characterization

Livers were pre-fixed in 2.5% glutaraldehyde solution for 24 h and then removed, rinsed in PBS buffer, and post-fixed for 2 h in 1% osmium acid. After rinsing in double-distilled water, the tissue was dehydrated sequentially in a series of alcohol concentrations, embedded in 100% resin, cut into 60–100 nm slices on an ultrathin microtome (Leica, EM-UC6, Wetzlar, Germany), retained in 3% uranyl acetate-lead citrate, and observed and photographed under a transmission electron microscope (Hitachi, H-7500, Tokyo, Japan).

#### 2.2.8. Statistical Analysis

Student’s *t*-test was used to compare the data between the NL and HL groups. One-way analysis of variance (ANOVA) followed by Tukey’s multiple-range tests were used for analyzing the data among different LBP levels in the HL diet. The linear or quadratic effect of LBP was assayed by orthogonal polynomial contrasts using the SPSS 27.0 software. All data are presented as the means ± standard deviation (SD). In all statistical tests used, *p* < 0.05 was considered significantly different. Data visualization was analyzed with the GraphPad Prim 9.0 (GraphPad Inc., La Jolla, CA, USA)

## 3. Results

### 3.1. LBP Improved the Growth Performance of Common Carp

As shown in Table 3, the FBW, WGR, and SGR in the HL group were significantly lower than those in the NL group (*p* < 0.05), whereas fish fed with the HL diet remarkably increased the FCR relative to fish fed with the NL diet (*p* < 0.01). The FBW, WGR, FI, and SGR increased linearly (*p* < 0.01) in response to increasing LBP levels. Notably, the FCR decreased both linearly and quadratically (*p* < 0.01) as LBP levels increased in the diet.

### 3.2. LBP Enhanced Antioxidant Capacities in the Liver

**Figure 1:**
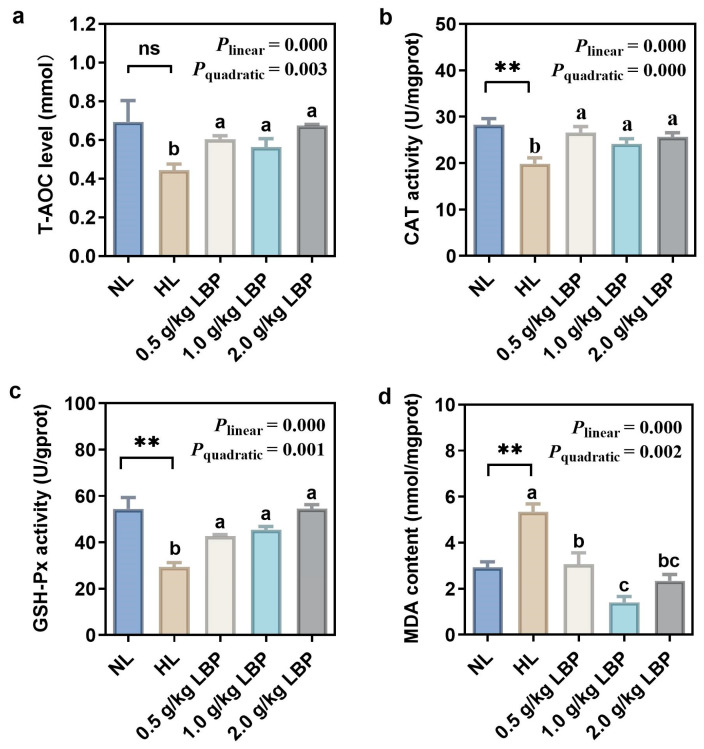
Effects of LBP supplementation on antioxidant capacities in the liver of common carp (*Cyprinus carpio*). Antioxidant enzymes include (**a**) total antioxidant capacity (T-AOC); (**b**) catalase (CAT); (**c**) glutathione peroxidase (GSH-Px); and (**d**) malondialdehyde (MDA). Data are presented as mean ± SD (n = 3). “*” represents statistical difference between the NL group and the HL group, using Student’s *t*-test (** *p* < 0.01). “ns” represents no significant difference. “abc” represents statistical difference among different LBP levels in the HL diet. *p*-values indicate a linear and quadratic decrease or increase in dietary LBP levels using orthogonal polynomial analysis.

The antioxidant capacities of common carp fed with LBP supplementation are presented in Figure 1. The CAT and GSH-Px activities in the HL group were significantly lower than those in the NL group (*p* < 0.01). Correspondingly, MDA content in the liver in the HL group was significantly increased (*p* < 0.01), whereas there was no significant difference in T-AOC activity between the NL and HL groups (*p* > 0.05). Dietary supplementation with LBP increased the T-AOC level and CAT and GSH-Px activities and decreased MDA content linearly and quadratically (*p* < 0.01 or *p* < 0.001).

### 3.3. LBP Decreased Hepatic Lipogenesis-Related Enzyme Activity

**Figure 2:**
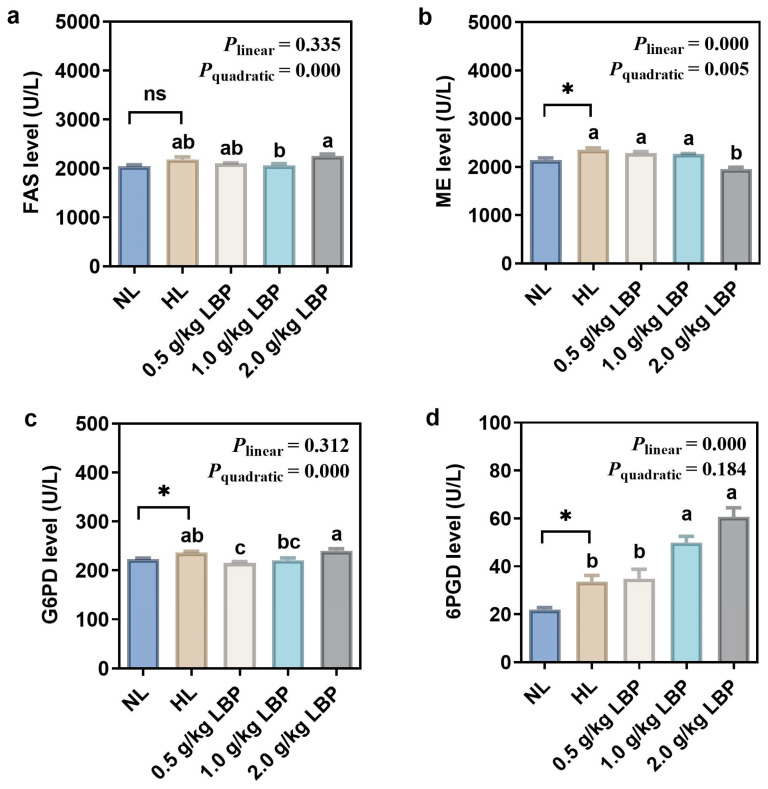
Effects of LBP supplementation on the lipid synthetase activity in common carp (*Cyprinus carpio*). Lipogenic enzymes include (**a**) fatty acid synthetase (FAS); (**b**) malic enzyme (ME); (**c**) glucose-6-phosphate dehydrogenase (G6PD); and (**d**) 6-phosphogluconate dehydrogenase (6PGD). Data are presented as mean ± SD (n = 3). “*” represents statistical difference between the NL group and the HL group, using Student’s *t*-test (* *p* < 0.05). “ns” represents no significant difference. “abc” represents statistical difference among different LBP levels in the HL diet. *p*-values indicate a linear and quadratic decrease or increase in dietary LBP levels using orthogonal polynomial analysis.

The hepatic liposynthetic enzyme activities of common carp fed with LBP supplementation are presented in Figure 2. The ME, G6PD, and 6PGD levels were significantly enhanced in fish fed with the HL diet compared to those in fish fed by the NL diet (*p* < 0.05), whereas there was no significant difference in FAS level between the NL and HL groups (*p* > 0.05). The FAS and G6PD activities decreased and then increased (*p* < 0.001) with increasing LBP levels, which was accompanied by a linear elevation (*p* < 0.001) in the 6PGD activity. The ME level both increased linearly (*p* < 0.001) and quadratically (*p* < 0.01) in response to increasing dietary LBP levels.

### 3.4. LBP Increased Hepatic Lipase Activity

**Figure 3:**
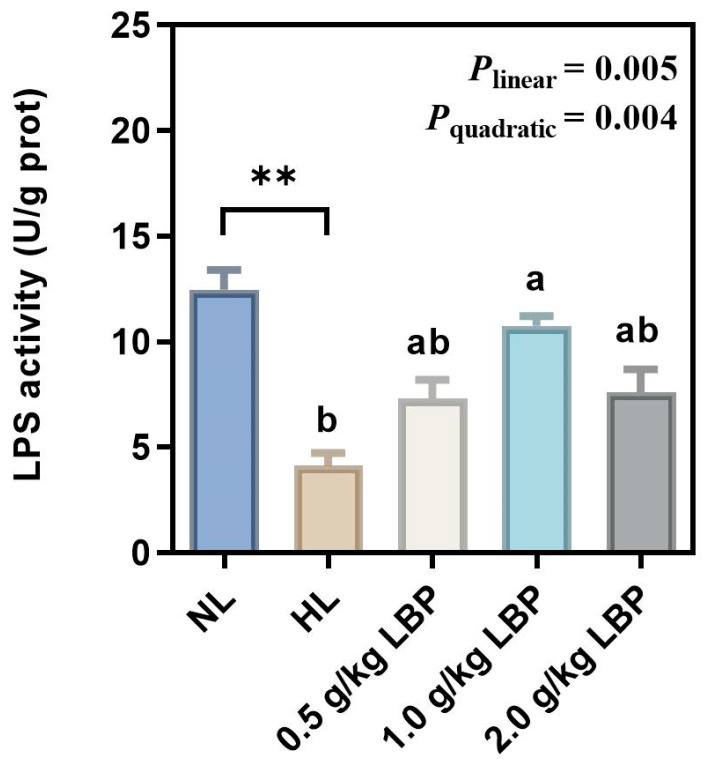
Effects of LBP supplementation on the lipase activity in common carp (*Cyprinus carpio*). Data are presented as mean ± SD (n = 3). “*” represents statistical difference between the NL group and the HL group, using Student’s *t*-test (** *p* < 0.01). “ab” represents statistical difference among different LBP levels in the HL diet. *p*-values indicate a linear and quadratic decrease or increase in dietary LBP levels using orthogonal polynomial analysis.

As presented in Figure 3, the LPS activity was significantly lower in the HL group than that in the NL group (*p* < 0.01). Dietary LBP linearly (*p* < 0.01) and quadratically (*p* < 0.01) promoted the LPS activity.

### 3.5. LBP Improved Hepatic Mitochondrial Function

**Figure 4:**
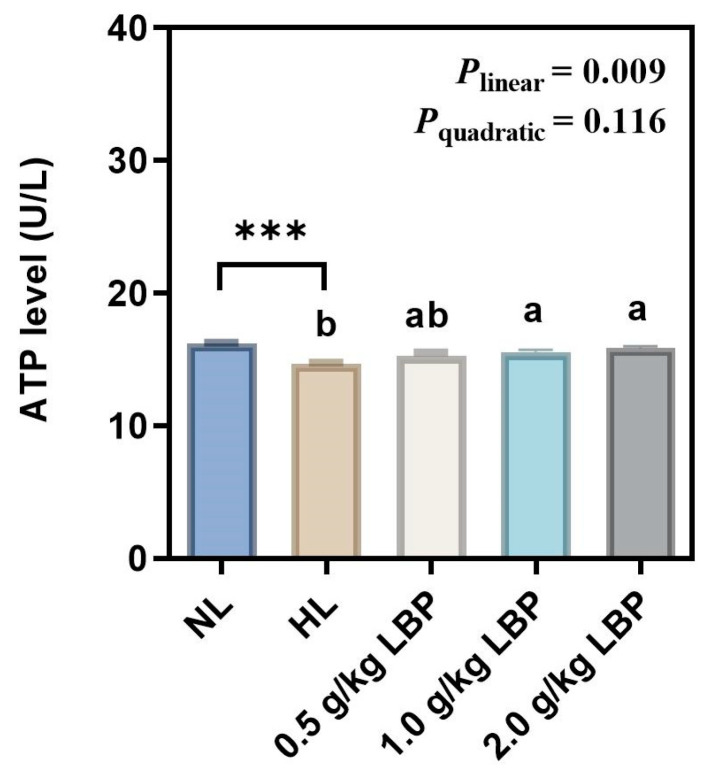
Effects of LBP supplementation on the ATP level in common carp (*Cyprinus carpio*). Data are presented as mean ± SD (n = 3). “*” represents statistical difference between the NL group and the HL group, using Student’s *t*-test (*** *p* < 0.001). “ab” represents statistical difference among different LBP levels in the HL diet. *p*-values indicate a linear and quadratic decrease or increase in dietary LBP levels using orthogonal polynomial analysis.

The ATP level in the liver of common carp fed with LBP supplementation is shown in Figure 4. The ATP level in the HL group was significantly decreased compared to that in the NL group (*p* < 0.001). Dietary LBP resulted in a linear (*p* < 0.01) elevation in the ATP level of common carp.

### 3.6. LBP Regulated The Expression of Genes Related to Lipid Metabolism

**Figure 5:**
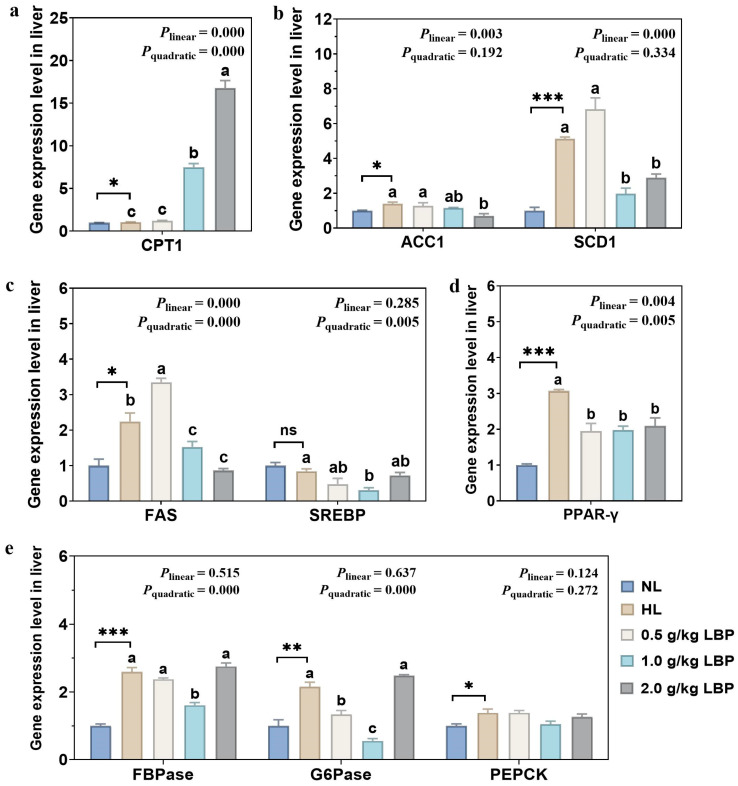
Effects of LBP supplementation on the lipid metabolism-related gene expression levels in common carp. (**a**) Fatty acid oxidation-related genes, including carnitine palmitoyltransferase 1 (*CPT*1); (**b**) fatty acid synthesis-related genes, including acetyl coenzyme A carboxylase 1 (*ACC*1) and stearoyl coenzyme A desaturase-1 (*SCD*1); (**c**) fat synthesis-related genes, including fat synthase (*FAS*) and sterol regulatory element-binding protein (*SREBP*); (**d**) lipolysis-related gene, including peroxisome proliferator-activated receptor-γ (*PPAR*-γ); and (**e**) gluconeogenesis-related genes, including fructofuranose bisphosphatase (*FBPase*), glucose-6-phosphatase (*G*6*Pase*), and phosphoenolpyruvate carboxykinase (*PEPCK*). Data are presented as mean ± SD (n = 3). “*” represents statistical difference between the NL group and the HL group, using Student’s *t*-test (* *p* < 0.05, ** *p* < 0.01 or *** *p* < 0.001). “ns” represents no significant difference. “abc” represents statistical difference among different LBP levels in the HL diet. *p*-values indicated a linear and quadratic decrease in dietary LBP levels using orthogonal polynomial analysis.

The lipid metabolism-related gene expression levels in the liver of common carp fed with LBP-supplemented diets are shown in Figure 5. Fish fed with the HL treatment significantly upregulated (*p* < 0.05 or *p* < 0.01 or *p* < 0.001) the mRNA expression levels of *CPT1*, *ACC1*, *SCD1*, *FAS*, *PPAR-γ*, *FBPase*, *G6Pase*, and *PEPCK* relative to fish fed with the NL treatment. Simultaneously, the mRNA expression levels of *FAS* and *PPAR-γ* both downregulated linearly and quadratically (*p* < 0.01 or *p* < 0.001), and the mRNA expression levels *of ACC1* and *SCD1* downregulated linearly (*p* < 0.01 or *p* < 0.001) in response to increasing LBP levels. In addition, a pronounced downregulation was observed for mRNA expression levels of *SREBP, FBPase*, and *G6Pase* with increasing LBP levels (quadratic effect, *p* < 0.01 or *p* < 0.001). Additionally, the mRNA expression levels of *CPT1* upregulated linearly and quadratically (*p* < 0.001) in response to increasing dietary LBP levels. However, there was no significant effect on *PEPCK* gene expression level with increasing LBP (*p* > 0.05).

### 3.7. Ultrastructural Images Revealed That LBP Alleviated Hepatic Lipid Deposition

**Figure 6:**
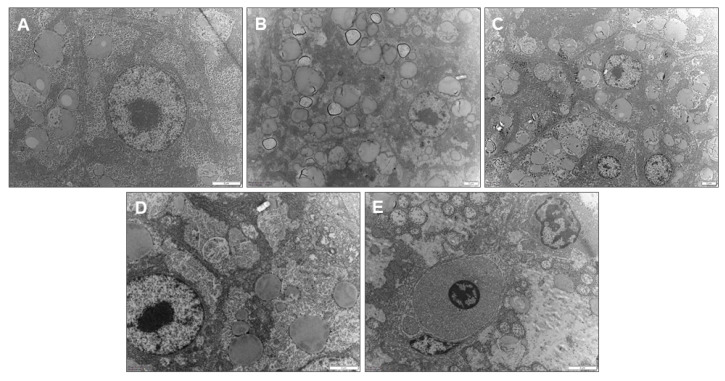
Transmission electron microscopy micrographs of common carp fed with different levels of LBP. (**A**) NL group; (**B**) HL group; (**C**) 0. 5 g/kg LBP group; (**D**) 1.0 g/kg LBP group; and (**E**) 2.0 g/kg LBP group. Scale bar of ultrastructure is 2 μm.

The hepatic ultrastructure of carp fed with different levels of LBP in HL diets is shown in Figure 6. Fish fed an NL diet had normal ultrastructure with rounded and well-defined nucleus, and fewer lipid droplets were observed. Compared with the NL group, the HL group exhibited deformed nucleus with blurred outlines and hypertrophied intracytoplasmic lipid droplets. Supplementation of 0.5–2.0 g/kg LBP gradually reduced lipid accumulation in the liver of the fish, and the number of lipid droplets around the central nucleus gradually became fewer and smaller.

## 4. Discussion

Growth performance has always been one of the focuses of greatest concern in practical production. Studies have confirmed that HL diets reduced the growth performance of blunt snout bream (*Megalobrama amblycephala*) [25], Nile tilapia [1], largemouth bass *(Micropterus salmoides)* [26], and grass carp [27]. In the present study, a significant decrease in FBW, WGR, and SGR was observed in common carp in the HL group compared to those in the NL group, indicating that the HL diet adversely affected the growth of carp. This may be related to the fact that the lipid intake of the fish exceeded their requirements, resulting in metabolic disorders, thus producing a decrease in growth performance [28]. Polysaccharides, as a kind of plant natural extract, are compounds consisting of multiple monosaccharides of the same or different structures combined through glycosidic bonds, which are characterized by high safety and low toxicity [29]. In aquatic animals, studies have demonstrated that dietary supplementation with 0.5–2.0 g/kg LBP significantly improved the growth performance of Turkestan barbel [17], spotted sea bass [18], Nile tilapia [22], and hybrid grouper [30]. Our results were similar to those mentioned above in that FBW, WGR, and SGR significantly increased with increasing LBP supplementation, and the optimal growth performance was achieved at 2.0 g/kg LBP supplementation. It has been shown that phytopolysaccharides promoted glucolipid metabolism and increased amino acid and protein synthesis by promoting the synthesis and secretion of growth-related hormones, thereby achieving a growth-promoting effect [31,32]. In parallel, it is noteworthy that the FI of carp increased significantly after supplementation with 2.0 g/kg LBP in the HL group. Nonetheless, it is understandable that there is some degree of correlation between FI and weight gain, which may also explain the better growth performance of carp after supplementation with 2.0 g/kg LBP.

Excessive lipid deposition in fish induces oxidative stress and generates large amounts of reactive oxygen species (ROS), which can cause oxidative damage to the organism if the antioxidant system fails to scavenge these free radicals in a timely manner [33]. Fish have developed an antioxidant enzyme defense system during the evolutionary process, including antioxidant enzymes such as superoxide dismutase, which can catalyze disproportionation of superoxide anion to generate O_2_ and H_2_O_2_, and H_2_O_2_ is converted to H_2_O and O_2_ under the synergistic effect of CAT and GSH-Px, thus protecting the organism from oxidative stress damage [34]. As an important product of lipid peroxidation, MDA can be used as a marker of the degree of oxidative stress [33]. In the present study, it was found that the MDA content in carp supplemented with HL diets was significantly elevated, whereas the T-AOC, CAT, and GSH-Px activities were significantly decreased. This is consistent with the results in turbot (*Scophthalmus maximus*) [35] and rainbow trout (*Oncorhynchus mykiss*) [36], suggesting that HL diets cause a decrease in antioxidant capacity and oxidative stress damage to the organism. LBP has a powerful antioxidant function, which can effectively scavenge free radicals and thus reduce oxidative stress damage in the organism. Studies have shown that LBP effectively increased the activities of antioxidant enzymes and inhibited the increase of MDA content in the livers of spotted sea bass [37] and zebrafish [38]. In the present study, supplementation of 0.5–2.0 g/kg LBP in the HL group significantly increased the activities of CAT and GSH-Px in the liver of carp and reduced the hepatic MDA content, thus enhancing the antioxidant capacity of carp. It has been reported that sturgeons fed with polysaccharides demonstrated significantly elevated CAT and GSH-Px activities and reduced MDA content [39]. Additionally, LBP supplementation in HL diets significantly elevated CAT and T-AOC activities and reduced MDA content [19], similarly to our findings, suggesting that LBP can enhance the antioxidant enzyme activities of carp, alleviate oxidative stress induced by HL diets, and thus maintain the hepatic redox homeostasis. T-AOC reflects the degree of peroxidative damage suffered by the organism to a certain extent and is a comprehensive indicator of the antioxidant capacity of the animal organism. Our data showed that hepatic T-AOC activity was significantly elevated after supplementation with different levels of LBP, which indicated that LBP elevated hepatic antioxidant capacity, and the elevation of T-AOC activity may be realized by increasing the activities of peroxidative catabolic enzymes, such as CAT and GSH-Px, to scavenge excessive reactive oxygen species.

G6PD, 6PGD, ME, and FAS synthesize fatty acids by providing NADPH and play an essential role in adipogenesis [40]. NADPH, as an electron donor, is involved in fatty acid, steroid, and DNA reductive biosynthesis, as well as oxidation–reduction reactions in organisms, and maintains cellular antioxidant defense system [41]. Our results showed that the levels of G6PD, 6PGD, and ME in the liver of common carp were significantly elevated after the carp were fed HL diets, which is consistent with the results of a previous study [42], suggesting that HL diets exacerbated lipogenesis in the liver of common carp. The role of polysaccharides in reducing lipogenesis has been demonstrated: it can regulate abnormalities in lipid metabolism by inhibiting fat synthesis through influencing the activity of lipogenesis enzymes [43,44,45]. It has been reported that polysaccharide could effectively reduce serum and intestinal lipogenesis-related enzyme activities, which suggests that polysaccharide can inhibit lipogenesis-related enzyme activities and thus inhibit lipogenesis [46]. In addition, polysaccharide was able to significantly reduce FAS levels in common carp [47]. Similar to these results, in our study, supplementation with 0.5–2.0 g/kg LBP in the NL diet reduced G6PD and ME levels, which suggested that LBP reduced lipogenesis in the liver of carp by decreasing lipogenesis-related enzyme activities. Concurrently, it also indicated that LBP promoted NADPH production, which may indirectly reflect that LBP supplementation increased the antioxidant capacity of carp, consistent with our results regarding antioxidant enzyme activities. Alternatively, LPS is a triacylglycerol acyl hydrolase that catalyzes the hydrolysis of natural substrate lipids and oils to produce fatty acids, glycerol, glycerol monoesters, or glycerol diesters [48]. In our study, LPS activity was significantly decreased in the HL group. Supplementation with 1.0 g/kg LBP significantly elevated liver LPS activity in carp, indicating that LBP accelerated lipolysis, thereby inhibiting lipid deposition. The TEM results visualized the lipid deposition within the hepatocytes more intuitively. In this experiment, there were abnormalities such as large lipid droplets, cytoplasmic vacuolization, nucleus deformation, and mitochondrial swelling in the hepatocytes of the HL group. After supplementation with LBP, smaller lipid droplets, rounded nucleus, homogeneous cytoplasm, and clearly visible cell membranes were observed in the hepatocytes. It was demonstrated that lipid deposition in the liver causes morphological abnormalities in the mitochondria and endoplasmic reticulum [49]. Mitochondria serve as the main site of fat β-oxidation [50], and their structural abnormalities inhibit lipolysis metabolism in mitochondria and exacerbate lipid accumulation in the liver. The endoplasmic reticulum is the site of lipoprotein synthesis, folding, assembly, and maturation [51], and changes in its structure would impede the normal synthesis and secretion of lipoproteins, thus leading to impaired fat transport. However, supplementation with LBP in the NL diet improved the hepatocyte abnormalities, and thus, it could be demonstrated that proper supplementation with LBP reduced hepatic lipid deposition, to a certain extent, and further influenced the internal structure, as well as the functioning, of the liver.

CPT-1 is one of the key enzymes involved in lipolytic metabolism. Mediated by PPAR-α, CPT1 catalyzes the conversion of lipoyl CoA to lipoyl carnitine, which initiates the β-oxidation of long-chain fatty acids in mitochondria [52]. Lu et al. [53] found that a 15% HL diet resulted in a significant downregulation of gene expression and enzyme activity of CPT1 in the liver of blunt snout bream. Similarly, NL diets have been reported to have downregulated CPT1 expression levels in the livers of channel catfish (*Ictalurus punctatus*) [54] and common carp [55]. PPAR-γ is mainly responsible for regulating lipid anabolism, enhancing gene expression of lipid synthesis-related enzymes, and promoting lipid deposition and adipocyte proliferation and differentiation in adipocytes. The transcription factor SREBP is a major regulator that initiates the de novo synthesis of fatty acids in the liver, and it is able to regulate lipid metabolism by modulating the synthesis of endogenous phospholipids and cholesterol. SREBP-1 is activated by plasma insulin under carbohydrate-sufficient conditions and upregulates the expression of genes for fatty acid synthesis-related enzymes (e.g., ACC and FAS, etc.) [56]. As found in grass carp [2] and channel catfish [54], a significant elevation of hepatic SREBP1 gene expression was induced by NL diets. It was also shown that supplementation with NL diets resulted in a significant increase in the mRNA expression levels of PPAR-γ and SREBP-1 in the liver [3]. SCD1 is a lipase that converts saturated fatty acids into monounsaturated fatty acids in hepatocytes and is a key regulator of fatty acid metabolic pathways [57]. ACC and FAS are the key enzymes involved in fatty acid synthesis. ACC catalyzes the carboxylation of acetyl-CoA to malonyl-CoA, and FAS converts malonyl-CoA to palmitic acid, which is ultimately esterified to triglycerides [58]. It has been demonstrated that NL diets significantly upregulate the hepatic expression levels of ACC and FAS in zebrafish (*Danio rerio*) [59]. Similarly, the expression level of FAS was also upregulated by NL diets in channel catfish [54]. Our results were similar to the above results, which suggested that lipid metabolism was impaired in the liver of fish induced by the NL diet, resulting in lipid deposition in the liver. LBP, as the main active ingredient in LBP, is known to ameliorate lipid metabolism by directly regulating the lipid metabolism pathway and thus improving the lipid metabolism of the organism. It was observed that LBP activated genes such as FAS and CPT1 and had a significant downregulation effect on the expression levels of SREBP-1,ACC1, and FAS in spotted sea bass [37], which was similar to our findings. It was recommended that fish inhibited lipogenesis by activating PPAR-γ to balance the potential lipid deposition induced by NL diets in the liver after supplementation with LBP. In addition, fish are capable of converting non-sugar substances into glucose and glycogen. Blood glucose levels are controlled by the liver through pathways such as gluconeogenesis and oxidation. PEPCK, FBPase, and G6pase are the rate-limiting enzymes in gluconeogenesis. PEPCK mainly catalyzes the generation of phosphoenolpyruvate from oxaloacetic acid. FBPase is responsible for catalyzing the conversion of fructose 1,6-diphosphate to fructose 6-phosphate, and G6paSe is able to catalyze the generation of glucose from fructose 6-phosphate. Our study showed that the expression levels of the rate-limiting enzymes *PEPCK*, FBPase, and *G*6*pase* were upregulated by the NL diet, and supplementation with LBP inhibited the expression levels of *FBPase* and *G*6*pase*, suggesting that LBP might alleviate NL diet-induced blood glucose elevation by inhibiting the gluconeogenic signaling pathway.

## 5. Conclusions

In conclusion, NL diets resulted in decreased growth performance, as well as oxidative stress injury, abnormal lipid metabolism, and liver histopathology in common carp. Supplementation with LBP (0.5–2.0 g/kg) in the NL diets promoted growth performance, enhanced antioxidant capacity, improved lipid metabolism, and reduced excessive lipid deposition in common carp under the NL diets.

## Figures and Tables

**Table 1:** Formulation and proximate composition of experimental diets (g/kg dry matter).

Ingredients	NL	HL	LBP0.05	LBP0.10	LBP0.20
Fishmeal	50	50	50	50	50
Chicken meal	50	50	50	50	50
Soybean protein concentrate	70	70	70	70	70
Soybean meal	350	350	350	350	350
Wheat middlings	270	270	270	270	270
Fish oil	20	70	70	70	70
Soybean oil	25	75	75	75	75
Vitamin premix ^1^	5	5	5	5	5
Trace mineral premix ^2^	5	5	5	5	5
Choline chloride	5	5	5	5	5
Dicalcium phosphate	20	20	20	20	20
Cellulose	7	7	6.5	6	5
Sodium carboxymethylcellulose	10	10	10	10	10
Lysine	6	6	6	6	6
Threonine	2	2	2	2	2
Methionine	5	5	5	5	5
LBP	0	0	0.5	1	2
Proximate compositions					
Crude protein (%)	30.24	30.02	30.35	30.08	30.14
Crude lipid (%)	5.93	15.92	15.68	15.76	15.88

^1^ The vitamin premix provided the following per kg of the diet: VA, 8000 IU; VC, 500 mg; VD_3_, 3000 IU; VE, 60 mg; VK_3_, 5 mg; VB_2_, 30 mg; VB_6_, 15 mg; VB_12_, 0.5 mg; choline chloride, 5000 mg; nicotinic acid, 175 mg; biotin, 2.5 mg; inositol, 1000 mg; folic acid, 5 mg; pantothenic acid, 50 mg. ^2^ The mineral premix provided the following per kg of the diet: Zn, 25 mg; Cu, 3 mg; Fe, 25 mg; Mn, 15 mg; I, 0.6 mg; Co, 0.1 mg; Se, 0.4 mg.

**Table 2:** The primers sequences used in the present study.

Gene	Primer Sequence (5′–3′)	GenBank Number
*CPT1* ^1^	F: CAGATGGAAAGTGTTGCTAATGAC R: TGTGTAGAAGTTGCTGTTGACCA	XM_051899001.1
*ACC1* ^2^	F: GTCACTGGCGTATGAGGATATT R:TCCACCTGTATGGTTCTTTGG	XM_042757417.1
*SCD1* ^3^	F: TTCGTCACCTTCAGCGCTAT R:CGCTTCTCTGGACACACGCT	XM_051917718.1
*FAS* ^4^	F: GACAGGCCGCTATTGCTATT R: TGCCGTAAGCTGAGGAAATC	XM_042767930.1
*SREBP* ^5^	F: CGTCTGCTTCACTTCACTACTC R:GGACCAGTCTTCATCCACAAA	XM_042752869.1
*PPAR-γ* ^6^	F: CTTCGTGAACC TGGACTTG R: ATCTGACCGTAGGAGATGAG	XM_042766485.1
*FBPase* ^7^	F: ACAGTCTGAATGAAGGCTAC R:CTCATACAACAGCCTCAGCT	XM_042756375.1
*G6Pase* ^8^	F: GCAGGTCAATCTCACTGGCT R:CTGATGTAGTGGAGCGCTAT	XM_042767111.1
*PEPCK* ^9^	F: GTCAGGTGCTGTGGCTGAAT R: TCCTTAGTGACAATCACAGT	XM_019080165.2
*β-actin*	F: GATCGGCAATGAGCGTTTCC R: ACGGTGTTGGCATACAGGTC	M24113.1

^1^*CPT1*, carnitine palmitoyltransferase 1. ^2^*ACC1*, acetyl coenzyme A carboxylase 1. ^3^*SCD1*, stearoyl coenzyme A desaturase 1. ^4^*FAS*, fat synthase. ^5^*SREBP,* sterol regulatory element-binding protein 1. ^6^ *PPAR-γ*, peroxisome proliferator-activated receptor-γ. ^7^ *FBPase*, fructofuranose bisphosphatase. ^8^ *G6Pase*, glucose-6-phosphatase. ^9^ *PEPCK*, phosphoenolpyruvate carboxykinase.

**Table 3:** Effects of *Lycium barbarum* polysaccharide (LBP) supplementation on growth performance of common carp (*Cyprinus carpio*) ^1^.

	NL	HL	0.5 g/kg LBP	1.0 g/kg LBP	2.0 g/kg LBP	*p*-Values ^2^	
						Linear	Quadratic
IBW ^3^ (g)	5.30 ± 0.20	5.33 ± 0.06	5.27 ± 0.15	5.27 ± 0.06	5.27 ± 0.15	0.521	0.631
FBW ^4^ (g)	41.23 ± 0.49	35.70 ± 2.29 ^b^*	40.93 ± 1.53 ^a^	41.00 ± 0.61 ^a^	43.63 ± 3.10 ^a^	0.002	0.314
WGR ^5^ (%)	676.40 ± 32.67	572.00 ± 50.80 ^b^*	676.77 ± 21.92 ^a^	677.43 ± 16.00 ^a^	727.50 ± 44.80 ^a^	0.001	0.230
FI ^6^ (g)	1431.06 ± 39.19	1400.62 ± 46.94 ^b^	1434.09 ± 48.52 ^b^	1445.47 ± 32.34 ^ab^	1561.99 ± 62.40 ^a^	0.004	0.178
SGR ^7^ (%/d)	3.79 ± 0.08	3.52 ± 0.14 ^b^*	3.80 ± 0.05 ^a^	3.80 ± 0.04 ^a^	3.91 ± 0.10 ^a^	0.001	0.166
FCR ^8^	1.59 ± 0.05	1.84 ± 0.08 ^a^**	1.61 ± 0.03 ^b^	1.62 ± 0.05 ^b^	1.63 ± 0.08 ^b^	0.005	0.009

^1^ Values expressed as mean ± SD, n = 3. “*” represents statistical difference between the NL group and the HL group, using Student’s *t*-test (* *p* < 0.05 or ** *p* < 0.01). “ab” represents statistical difference among different LBP levels in the HL diet. Values in the same row with different superscripts indicate significant difference (*p* < 0.05). ^2^ *p*-values indicate a linear and quadratic decrease or increase in dietary LBP levels using orthogonal polynomial analysis. ^3^ IBW, initial body weight. ^4^ FBW, final body weight. ^5^ WGR, weight gain rate. ^6^ FI, feed intake. ^7^ SGR, specific growth rate. ^8^ FCR, feed conversion ratio.

## Data Availability

Data are contained within the article.
